# Hearing Health Healthcare Disparities in Appalachia

**DOI:** 10.13023/jah.0304.02

**Published:** 2021-10-25

**Authors:** Matthew L. Bush

**Affiliations:** Department of Otolaryngology – Head and Neck Surgery, University of Kentucky Medical Center

**Keywords:** Appalachia, hearing loss, audiology, rural health services, health disparities

## Abstract

Hearing loss is a global public health issue with disproportionate negative impacts on those who live in rural regions, such as Appalachia. This commentary provides an overview of hearing health and healthcare disparities in rural regions along with discussion of the significance of recent research findings which highlight the incidence of hearing loss and the shortage of hearing specialists in Appalachia.

Hearing loss is a major global public health problem. According to the 2021 World Report on Hearing, approximately 1.5 billion people, including adults and children, worldwide have hearing loss.^1^ It currently represents the third most prevalent chronic health condition among adults within the U.S.^2^ Children with hearing loss, either of congenital or acquired causes, are at risk for delayed language development.^3^ Undiagnosed and untreated hearing loss causes a negative impact on the social, occupational, cognitive, and emotional well-being of those affected.^4^,^5^ There is a significant global financial impact, as well, with nearly $980 billion in yearly economic losses due to hearing loss.^1^ Unfortunately, as is the case with many chronic diseases, the global trends in hearing loss indicate that the greatest prevalence and burden of disease occurs in regions with the least accessible healthcare. The research presented in this issue of the *Journal of Appalachian Health* by Pudrith et al.^6^ illustrates that trend by focusing our attention on the hearing health and healthcare status of the Appalachian region of the U.S., which has suffered from pervasive and persistent health disparities.

Hearing loss is medical condition that involves multi-specialty hearing healthcare specialists such as otolaryngologists and audiologists and may be managed with a variety of medical, surgical, and technological treatments. The lack of hearing healthcare access, utilization, and affordability continues to play a major role in preventing timely diagnosis and treatment of hearing loss. The inaccessibility and under-utilization of hearing healthcare is amplified in vulnerable populations, such as rural adults. Approximately 20% of the US population reside in rural areas and rural Americans tend to be poorer, less educated, suffer higher rates of disease and disability, and are more likely to be uninsured than their urban counterparts.^7^,^8^ Rural adults face a greater risk of death from major diseases; however, these rural residents may have the most limited access to and poorest utilization of healthcare.^9^ Systemic factors such as lack of public transportation, lack of telecommunications access, fewer community services, and a shortage of healthcare providers contribute to sub-optimal health among rural Americans.^8^ Hearing loss is more prevalent among adults living in rural communities compared with those in urban settings.^10^,^11^ Awareness of benefits from diagnosis of hearing loss and treatment is low among the rural population and among healthcare professionals.^12^,^13^ Screening for hearing loss among adults is uncommon in the primary care setting and there is poor adherence to recommended treatment.^14^,^15^ There is also a critical shortage of hearing healthcare specialists within rural areas.^16^ While these features are common in much of rural America, rural Appalachia possesses an extreme version of these health disparity and care inequity characteristics.^8^ Appalachia is an expansive and diverse region encompassing over 25 million people throughout 13 states.^17^ Across the lifespan, rural Appalachian adults with hearing loss have faced delayed diagnosis and treatment, lack of treatment, and poorer adherence/utilization of evidence-based treatments.^18^,^19^

Against this backdrop of hearing loss significance and long-standing rural hearing healthcare disparities, this manuscript presents novel findings regarding hearing loss prevalence and audiological disparities in Appalachia. Much of what we know about rural hearing loss prevalence and accessibility to hearing healthcare specialists in rural regions is based on work outside the Unites States or in non-Appalachian regions of the U.S. This manuscript utilizes a combination of population databases to conduct their univariate and multivariate analysis comparing Appalachian regions with non-Appalachian regions. The authors have quantified the higher incidence of subjective hearing loss among Appalachian residents and through their multiple linear regression analyses have presented data that demonstrates that Appalachian residence, apart from rural residence, is independently associated with a higher incidence of hearing loss. Furthermore, their pairwise linear regression data provides us with a deeper understanding of the disparity at hand by quantifing that relationship between rurality, prevalence of hearing loss, and the shortage of audiologists. While we neither understand the causes underlying this health disparity nor the solution to this problem, this study gives us clearly articulated evidence that helps us understand this disparity better. This data pushes us to consider how specialty care can be delivered to vulnerable populations in remote locations. Considerable innovation is needed to address rural and Appalachian hearing health and healthcare disparities. New models and methods for care delivery are needed to explore how to deliver specialty healthcare when there are no or very few specialists nearby. Research related to hearing loss may be less familiar for the readers of this journal; however, I encourage readers to consider the importance of work such as this to define and describe a complex health disparity issue within a resource-limited region such as Appalachia.
